# Apelin – A New Kid on the Block in Periodontology

**DOI:** 10.3290/j.ohpd.b5695264

**Published:** 2024-08-27

**Authors:** Pablo Cores Ziskoven, Andressa V. B. Nogueira, Onur Yoldaş, Nurcan Buduneli, Philipp S. Wild, Thomas Koeck, James Deschner

**Affiliations:** a Dentist, Department of Periodontology and Operative Dentistry, University Medical Center of the Johannes Gutenberg University, Mainz, Germany. Study conception and design, literature search, data analysis and interpretation, prepared the original draft, edited the manuscript, visualisation, reviewed and approved the final version of the manuscript.; b Dentist, Department of Periodontology and Operative Dentistry, University Medical Center of the Johannes Gutenberg University, Mainz, Germany. Study conception and design, literature search, data analysis and interpretation, prepared the original draft, edited the manuscript, visualisation, supervision, reviewed and approved the final version of the manuscript.; c Dentist, Department of Periodontology, School of Dentistry, Ege University, İzmir, Turkey. Study conception and design, literature search, prepared the original draft, edited and reviewed the manuscript, reviewed and approved the final version of the manuscript.; d Professor, Department of Periodontology, School of Dentistry, Ege University, İzmir, Turkey. Study conception and design, literature search, prepared the original draft, edited and reviewed the manuscript, reviewed and approved the final version of the manuscript.; e Professor, Preventive Cardiology and Preventive Medicine, Center for Cardiology, University Medical Center of the Johannes Gutenberg University Mainz, Mainz, Germany; Clinical Epidemiology and Systems Medicine, Center for Thrombosis and Hemostasis, University Medical Center of the Johannes Gutenberg University Mainz, Mainz, Germany; Institute of Molecular Biology (IMB), Mainz, Germany; German Center for Cardiovascular Research (DZHK), Partner Site Rhine-Main, Mainz, Germany. Study conception and design, literature search, prepared the original draft, edited and reviewed the manuscript, supervision, reviewed and approved the final version of the manuscript.; f Head of Targeted Proteomic Biomarker Laboratory, Preventive Cardiology and Preventive Medicine, Center for Cardiology, University Medical Center of the Johannes Gutenberg University Mainz, Mainz, Germany. Study conception and design, literature search, prepared the original draft, edited and reviewed the manuscript, visualisation, supervision, reviewed and approved the final version of the manuscript.; g Professor, Department of Periodontology and Operative Dentistry, University Medical Center of the Johannes Gutenberg University, Mainz, Germany.Study conception and design, literature search, data analysis and interpretation, prepared the original draft, edited and reviewed the manuscript, visualisation, supervision, reviewed and approved the final version of the manuscript.

**Keywords:** apelin, adipocytokine, adipokine, periodontitis, periodontium

## Abstract

Periodontitis is associated with numerous systemic diseases, and it has been shown that these associations are partly causal in nature. It is assumed that such interactions between periodontal and systemic diseases are also mediated via adipokines. Apelin, an adipokine about which there is little research in the dental field, is also produced together with its receptor in periodontal cells. The aim of this review was to summarize the currently available literature on the apelin-APJ system to better understand the pathomechanistic relationship between periodontitis and obesity and to determine the potential clinical relevance of apelin for diagnostics and therapy. In vitro studies suggest that apelin can enhance bacterial-induced synthesis of proinflammatory and proteolytic molecules, indicating a significant etiopathogenic role of this adipokine. Since serum levels of apelin are elevated in diabetes and/or obesity, it is possible that such systemic diseases promote the development and progression of periodontitis via apelin. On the other hand, it is also conceivable that apelin from the periodontium influences such systemic diseases. Further research is needed to better understand the role of apelin in the periodontium and the entire oral cavity, but also in the interactions between periodontal and systemic diseases. In particular, clinical intervention studies are needed to further decipher the etiopathogenic role of apelin in periodontitis.

## Periodontitis and its Link to Systemic Diseases

Periodontitis is a chronic inflammatory disease of the periodontium characterized by irreversible bone and attachment loss.^62^ If this disease remains untreated, it can lead to tooth loss, reduced quality of life and negative effects on the entire organism. Periodontitis is caused by microbial dysbiosis associated with an inadequate immune and inflammatory response.^10^ Microorganisms on the root surface can cause periodontitis directly, but more importantly indirectly by triggering an immunoinflammatory response in the periodontal tissues to reduce the microbial attack. If the inflammatory processes persist for too long and/or are too intense and/or misdirected, the disease becomes chronic and the periodontal soft and hard tissues are destroyed. There is clear evidence that a number of risk factors such as smoking, genetic and epigenetic predisposition, psychological stress and occlusal overload or misload can contribute to the development and progression of periodontitis.^38,53,63^ Various systemic diseases or conditions such as diabetes mellitus, obesity, and metabolic syndrome have also been shown to increase the risk.^5,36,52,54,66^ On the other hand, periodontitis may also promote the development and progression of these and other systemic diseases and conditions, meaning that there are not only significant associations between periodontitis and numerous systemic diseases, but also causal relationship at least in part, and this causality is even bidirectional. The aim of this review was to summarize the currently available literature on the apelin-APJ system to better understand the pathomechanistic relationship between periodontitis and obesity and to determine the potential clinical relevance of apelin for diagnostics and therapy.

## Adipokines

Several possible pathomechanisms have been described, and adipokines may play an important role in the link between periodontitis and metabolic diseases.^18,36,88^ A variety of immunoinflammatory but also structural cells of the periodontium, e.g. periodontal ligament (PDL) cells, osteoblasts, gingival fibroblasts and epithelial cells, as well as a large number of inflammatory mediators such as cytokines are involved in the etiopathogenesis of periodontitis. A special group of cytokines are adipokines (adipocytokines). It was originally assumed that these cytokines were only produced by adipocytes, hence named after these cells. However, it later became clear that such adipokines are also produced by other cells and tissues, where they exert physiological and pathophysiological significance.^18,25^ Thus, adipokines represent a pathomechanistic link between obesity and systemic diseases because adipose tissue is not only a pure energy store but also an active metabolic organ, whose secretion of adipokines is dysregulated when excessive.^61^ These adipokines can regulate glucose, fat, and bone metabolism as well as angiogenesis, play an important role in the development of cancer, influence the sense of hunger and/or thirst, but can also exhibit pro- or anti-inflammatory effects.^15,23,43^

### Presence and Role of Adipokines in Periodontium

Interestingly, the presence of such adipokines has also been demonstrated in the periodontium, suggesting that such adipokines may also play an important role in the development and progression of periodontitis.^16–18,37,55,56,59,77^ In recent years, the role of adipokines such as visfatin,^17,55^ resistin,^2,56^ leptin,^59,69^ and adiponectin^58,89^ in the periodontium has been increasingly studied. In PDL cells, for example, visfatin (nicotinamide phosphoribosyltransferase, pre-B-cell colony-promoting factor) has been shown to increase the expression of matrix metalloproteinase (MMP) 1 and chemokine C-C motif ligand (CCL) 2.^57^ Leptin has been shown to enhance tumor necrosis factor (TNF)-α gene expression.^67^ Resistin is another adipokine with proinflammatory properties, as it induces the expression of proinflammatory cytokines in the periodontium.^19,56^ In contrast, adiponectin was able to counteract on the stimulated increase in the expression of proinflammatory mediators such as interleukin (IL)-1β, IL-6, IL-8, MMP1 and -3 in PDL cells, suggesting an anti-inflammatory effect.^41,58^ Overall, there is fundamental evidence that resident cells of the periodontium and immunoinflammatory cells can produce adipokines and several of these adipokines exert proinflammatory and proteolytic effects on periodontal tissues, thereby contributing to periodontal inflammation and destruction seen in periodontitis.

### Apelin and its Receptor

Apelin was first isolated and described by Tatemoto et al. in 1998,^73^ while a G protein-coupled receptor was discovered in humans with a gene locus on chromosome 11 locus 11q12,^60^ which turned out to be the endogenous apelin receptor (APJ), was already described in 1933. The gene Xq25-q26.3 encodes the protein biosynthesis for preproapelin, a polypeptide of 77 amino acids which is converted into the 55 residue proapelin by removing the N-terminal signal peptide.^44^ At least four primary bioactive subforms can be formed from proapelin by single- or multi-stage proteolytic cleavages that are still not fully understood, with the C-terminus being retained: Apelin-36, -17, -13, and the N-terminal pyroglutamate-modified Pyr(1)-apelin-13.^64^ The proprotein convertase subtilisin/kexin type 3 (PCSK3) / Furin has been shown to directly convert proapelin into apelin-13.^70^ The receptor-binding C-terminus of these peptides is highly conserved across species in humans as well as in rats, mice, and cows. Rapid further proteolytic degradation determines duration and biological effectiveness of their action, with Pyr(1)-apelin-13, which is detected in human plasma, being the most bioactive subform.^64^ C-terminal cleavage of apelin-13 and Pyr(1)-apelin-13 by angiotensin converting enzyme 2 (ACE2) and at a lower rate by prolyl carboxypeptidase (PRCP) results in an still biologically active apelin-12, while proteolysis by the metalloproteinase neprilysin (NEP) results in apelin-7 and -8, which are biologically inactive.

## Role of Apelin in Health and Disease

Apelin was first isolated in tissues of the central nervous system, and the molecule was thought to play an essential role in central signal transduction.^44^ Over time, the apelin-APJ complex was discovered in virtually all organs, notably the cerebellum, vascular endothelium, heart, lungs, kidneys, liver, adrenal glands and adipose tissue,^32,39^ indicating that apelin has a broad spectrum of physiological effects, including regulation of metabolism, inflammation and apoptosis.^65,78^ Studies have shown differential expression of apelin in different tissues: low expression in kidney, liver and pancreatic tissue, medium expression in skeletal muscle and high expression in chondrocytes, endothelial cells, skin, brain, spleen, thymus, lung, and also in adipose tissue.^22^ The fact that apelin is secreted by adipocytes and thus falls under the definition of an adipokine was first confirmed by a research group in 2005.^13^ With regard to periodontal diseases, the demonstrated involvement of the apelin-APJ axis, particularly apelin-13, in the regulation of bone metabolism, the influence on apoptosis, proliferation and differentiation of osteoblasts in animal and human models, and the response of macrophages may be of potential relevance.^26,72,81^ In the heart, apelin is predominantly expressed constitutively in endocardial and vascular endothelial cells, while the APJ receptor is also expressed in vascular smooth muscle cells and cardiomyocytes.^40^ In isolated rat hearts, apelin induced a potent dose-dependent, saturable positive inotropic effect,^71^ and at least apelin-36, apelin-13, and Pyr(1)-apelin-13 lower blood pressure through peripheral vasodilation via nitric oxide (NO) secretion.^35,44^ It has been observed that apelin-13 promotes angiogenesis in animal models following myocardial infarction through increased proliferation, migration, and induction of vascular endothelial growth factor (VEGF) in endothelial cells.^87^ The apelin-APJ system further exerts anti-inflammatory and anti-fibrotic actions in part by counteracting the actions of the renin-angiotensin system and involved in the molecular actions of statins.^14^ Loss of apelin after myocardial infarction is related to a proinflammatory response (TNF-a, interleukin 1b), increased macrophage infiltration and infarct size, adverse remodeling, aggravated systolic dysfunction, and heart failure.^74,76,78^ Deficiency or overabundance of apelin-APJ signaling, particularly by apelin-13, is not only involved in inflammatory cardiovascular dysfunctions but also inflammatory processes in other organs.^78^ In lung tissue, apelin-13 stimulates alveolar formation and reduces inflammatory parameters.^75^

**Fig 1:**
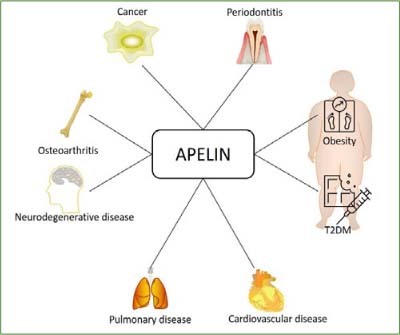
Association of apelin with different systemic diseases.

Effects of the apelin-APJ system on energy metabolism extend to both glucose and lipid metabolism including the modulation of insulin secretion^12,33^ and therefore plays a key role in metabolic diseases such as type 2 diabetes mellitus (T2DM) and obesity (Fig 1).^46^

In regard of glucose metabolism, decreased glycemia observed in wild type and insulin-resistant, obese mice injected with apelin-13 was mainly due to an increase in glucose utilization additive to insulin in white adipose tissue and skeletal muscles dependent on the energy sensor adenosine monophosphate dependent kinase (AMPK), Akt, and endothelial nitric oxide synthase (eNOS).^20^ Insulin-stimulated glucose uptake was also increased in insulin-resistant 3T3-L1 adipocytes by Pyr(1)-apelin-13^90^ and apelin-13 stimulated AMPK-dependent glucose uptake also in human adipose tissue ex vivo.^7^ Similar findings have been demonstrated in other tissues: In the intestinal tract^21^ and cardiac muscle cells,^82^ Pyr(1)-apelin-13 induced glucose uptake via GLUT2 and GLUT4, respectively. It has been shown that serum and plasma levels of apelin are increased in patients with T2DM^28,34,50^ and the adipokine has an impact on blood glucose regulation via positive modulation of glucose transport in different cell types^86,90^ and enhancement of insulin sensitivity.^1,4^ Based on these observations and the properties of apelin, it has been speculated to make a physiological compensation for insulin deficiency.

Only a few studies describe the effects of apelin on lipid metabolism. While there are conflicting results regarding the influence on lipolysis in mice and humans, apelin stimulates utilization of lipids.^12^ Apelin-13 was shown to AMPK-dependent increase fatty acid oxidation, oxidative capacity, and mitochondrial biogenesis in skeletal muscle of high-fat diet induced obese and insulin-resistant mice.^8^ In addition, apelin-overexpressing transgenic mice showed resistance to obesity related to increased lymphatic and blood vessel integrity preventing vascular permeability related fatty acid uptake in adipose tissue and increased blood vessel formation in skeletal muscle.^83^ Accordingly, apelin knockout mice showed excessive weight gain. Elevated apelin concentrations in human serum have also been found in obesity,^9,42^ but normalized with dietary changes and calorie reduction.^12^

It therefore seems likely that the metabolic effects induced by elevated plasma apelin concentrations in metabolic diseases such as obesity and T2DM are, at least initially, part of a compensatory mechanism to maintain functions such as insulin sensitivity.

In addition to its physiological effects, apelin is also involved in pathological processes such as osteoarthritis and cancer.^79,84^ Data on the role of the apelin/APJ system in cancer vary widely depending on apelin subform and tissue type.^6,80^ However, the adipokine clearly has a modulatory influence on various cancers such as prostate,^48^ gastric,^24^ ovarian,^31^ lung,^11^ and oral squamous cell carcinomas.^29^ Pathomechanistic explanations could be apelin-induced increased cell migration in various tumor tissues,^24,29,49^ increased expression of pro-angiogenic factors in tumor cells and/or apelin-stimulated proliferation of endothelial cells in tumor tissues.^3^

## Presence and Potential Role of Apelin in Periodontium

The three-way molecular pathological interplay in the chronic multimorbidity of periodontitis, diabetes and obesity, which is maintained by common biological mechanisms such as inflammation and insulin resistance, is of great importance to dentists.^52^ The energy metabolism modulation by proinflammatory and antiinflammatory effects of adipokines such as leptin, adiponectin, resistin, visfatin, and vaspin are also part of this molecular pathological interplay.^27,47,68^ Based on the positive or negative correlations observed between molecular and clinical status, which are also shown in the positive correlation of the severity of diabetes and periodontitis, increased or decreased adipokine levels in body fluids could both be an indication of the severity of these metabolic diseases and chronic periodontitis and also mutually promote the development and progression of such multimorbid status. Poorly controlled diabetics have a threefold increased risk of periodontitis, and severe periodontitis can further worsen the derailed metabolic situation. Moreover, successful periodontal therapy reduces HbA1c levels in type 2 diabetes mellitus (T2DM) by an average of 0.4 %, demonstrating that the relationship between periodontitis and diabetes is causal in nature.^66^ The increased serum/plasma concentrations of the adipokine apelin in diabetes and obesity^46^ are therefore also of interest. Recently, Hirani et al^30^ investigated the serum levels of apelin in periodontally and systemically healthy individuals and in periodontitis patients with and without T2DM. The study showed that apelin concentrations were significantly higher in the periodontitis group than in the healthy control group. Furthermore, apelin concentrations were highest in patients suffering from both periodontitis and T2DM suggesting that apelin plays a role in inflammation and glucose regulation. Sarhat et al. examined the apelin concentration in the saliva of periodontally diseased diabetics as well as periodontally and systemically healthy individuals.^68^ They also found the highest apelin concentrations in patients with periodontitis and T2DM. The increased systemic apelin concentrations in diabetes and obesity may be explained by its compensation for the reduced insulin sensitivity in T2DM. However, it is possible that resistance/tolerance to apelin develops over a longer period of time, as is also known for leptin in metabolic diseases.^51^ The consequence would then be an increased apelin level, as is the case for insulin. Such sustained elevation of apelin levels may negatively affect the periodontium. Our research group has recently demonstrated for the first time that apelin concentrations are not only altered systemically in serum and saliva, but also locally in gingival crevicular fluid (GCF) samples.^85^ We compared the GCF apelin levels of individuals with clinically healthy periodontal tissues with those of patients suffering from gingivitis or periodontitis, all of whom were systemically healthy. We found that periodontitis patients had the highest GCF apelin levels, followed by gingivitis patients and healthy controls. This suggests that apelin may have a potential as a diagnostic biomarker in periodontology. Systemically elevated apelin concentrations have also been found in association with other systemic diseases.^6^ Future studies are warranted to clarify whether increased systemic apelin concentrations in these systemic diseases are also reflected in GCF, gingival tissue, and saliva samples.

**Fig 2:**
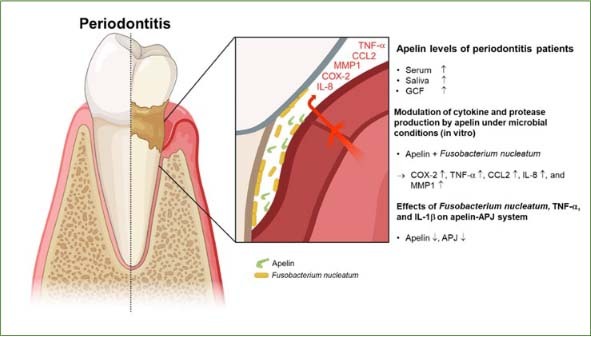
Summary of studies on the role of apelin in periodontal tissues and periodontitis.

To date, very little is known about the effects of apelin subforms on periodontal cells. We have recently addressed this issue in numerous in vitro experiments.^16^ As expected, the periodontopathogen *Fusobacterium nucleatum* (*F. nucleatum*) caused an upregulation of molecules associated with periodontal inflammation and destruction in our experiments. Interestingly, adding proteolytically unprocessed preproapelin increased *F. nucleatum*-induced expressions of CCL2 and MMP1 at 24 h and of COX2, CCL2, IL-8, TNF-α and MMP1 at 48 h. In addition, preproapelin was able to significantly increase the inhibition of RUNX2 induced by *F. nucleatum* at 24 h. Preproapelin alone had no significant effect on the expression of the above molecules indicating *F. nucleatum* dependent processing of preproapelin. These in-vitro results suggest that certain proteolytically processed apelin subforms may contribute to periodontal inflammation and tissue destruction by periodontopathogens. Elevated concentrations of certain apelin subforms in systemic diseases could therefore represent a pathomechanistic link for the association between periodontitis and certain systemic diseases. Moreover, our in vitro experiments showed that apelin and its receptor are constitutively expressed in PDL cells and that their spontaneous expression is also regulated by *F. nucleatum*. In a study by Lee et al,^45^ incubation of PDL cells and gingival fibroblasts with the inflammatory mediator TNF-α led to a downregulation of apelin. Our findings together with those of Lee et al^45^ suggest that the local apelin-APJ system in the periodontium is downregulated, at least initially, during periodontal microbial dysbiosis and inflammation (Fig 2). As dysregulation of the apelin-APJ system tends to have proinflammatory effects, the initial downregulation of apelin and its receptor may represent a transient, protective host compensatory mechanism to limit inflammation and associated tissue destruction. Nevertheless, the experiments also showed that this potentially protective inhibition of apelin and its receptor was no longer present after a certain period of time, suggesting that the apelin-APJ system may play a crucial role in the pathogenesis of periodontitis during prolonged periodontal inflammation. However, the initial downregulation could also be the result of either dysregulation (deficiency/overabundance) of the apelin-APJ system or the distribution of apelin forms due to altered processing or maybe both.

Further studies are required to clarify whether periodontal therapy alters apelin levels and its proteolytic processing in GCF, saliva, gingiva and serum. In that case, apelin subforms could serve as biomarkers for diagnostic purposes, as has been suggested for T2DM. In the early stages, periodontal diseases usually do not show severe subjective complaints or clinical symptoms, which often delays the patient’s visit to a specialist. However, an early diagnosis would be extremely important, as treatment at this point is efficient to avoid consequences of the disease. Probing pocket depth, clinical attachment loss, bleeding on probing, gingival index and plaque index are the most important clinical periodontal parameters and, together with radiographic assessment of alveolar bone loss, form the current basis of clinical periodontal diagnosis. Given the episodic nature of periodontal disease, these diagnostic tools are inadequate. Furthermore, clinical signs of tissue destruction provide information about the past, but only very limited information about the current status of periodontal disease activity. One of the challenges in periodontal diagnostics is to find a reliable clinical and/or biochemical biomarker that provides a valid indication of ongoing and/or future tissue destruction. Such a biomarker could enable dental care providers to offer individually tailored treatment plans and adjust the frequency of recalls during supportive periodontal therapy. Modulating the apelin-APJ system could lead to new immunomodulatory strategies in the treatment of periodontitis.

## Summary

**Fig 3:**
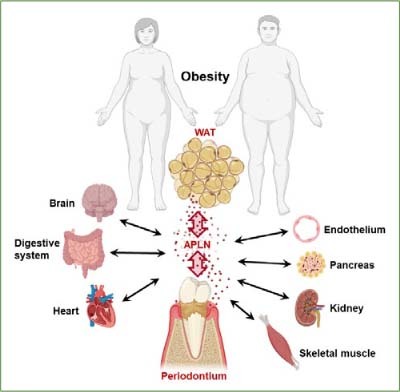
Schematic of apelin-based multidirectional organ interactions between periodontitis and obesity. WAT: white adipose tissue; APLN: apelin.

Periodontitis is associated with numerous systemic diseases, and it has been shown that these associations are partly causal in nature. It is assumed that such interactions between periodontal and systemic diseases are also mediated via adipokines. Apelin is an adipokine that has been little studied in relation to periodontitis to date. This adipokine is produced together with its receptor in periodontal cells. In vitro studies suggest that apelin can enhance bacterial-induced synthesis of proinflammatory and proteolytic molecules, indicating a significant etiopathogenetic role of this adipokine. Since the serum levels of apelin are elevated in diabetes and/or obesity, it is possible that these systemic diseases promote the development and progression of periodontitis via apelin. On the other hand, it is also conceivable that apelin from the periodontium influences such systemic diseases (Fig 3). Further research is needed to better understand the role of apelin subforms in the periodontium and the entire oral cavity, but also in the interactions between periodontal and systemic diseases. In particular, clinical intervention studies are needed to further decipher the etiopathogenetic role of apelin subforms in periodontitis. Further basic research is also needed on the regulatory effects of apelin subforms on periodontal wound healing and soft and hard tissue metabolism. A better understanding of the apelin-APJ system could pave the way for new diagnostic and therapeutic strategies in the treatment of periodontitis.
